# Severe congenital microcephaly with AP4M1 mutation, a case report

**DOI:** 10.1186/s12881-017-0412-9

**Published:** 2017-05-02

**Authors:** Sarah Duerinckx, Helene Verhelst, Camille Perazzolo, Philippe David, Laurence Desmyter, Isabelle Pirson, Marc Abramowicz

**Affiliations:** 1 IRIBHM, Université Libre de Bruxelles, Brussels, Belgium; 2 Department of Paediatric Neurology, Ghent University Hospital, Ghent, Belgium; 3 Department of Medical Genetics, Hôpital Erasme – Université Libre de Bruxelles, Brussels, Belgium; 4 Department of Medical Imaging and Radiology, Hôpital Erasme – Université Libre de Bruxelles, Brussels, Belgium

**Keywords:** Exome sequencing, Brain development, Consanguinity, Intellectual disability, Case report

## Abstract

**Background:**

Autosomal recessive defects of either the B1, E1, M1 or S1 subunit of the Adaptor Protein complex-4 (AP4) are characterized by developmental delay, severe intellectual disability, spasticity, and occasionally mild to moderate microcephaly of essentially postnatal onset.

**Case presentation:**

We report on a patient with severe microcephaly of prenatal onset, and progressive spasticity, developmental delay, and severe intellectual deficiency. Exome sequencing showed a homozygous mutation in *AP4M1*, causing the replacement of an arginine by a stop codon at position 338 of the protein (p.Arg338X). The premature stop codon truncates the Mu homology domain of AP4M1, with predicted loss of function. Exome analysis also showed heterozygous variants in three genes, *ATR, MCPH1* and *BLM*, which are known causes of autosomal recessive primary microcephaly.

**Conclusions:**

Our findings expand the AP4M1 phenotype to severe microcephaly of prenatal onset, and more generally suggest that the AP4 defect might share mechanisms of prenatal neuronal depletion with other genetic defects of brain development causing congenital, primary microcephaly.

**Electronic supplementary material:**

The online version of this article (doi:10.1186/s12881-017-0412-9) contains supplementary material, which is available to authorized users.

## Background

Primary microcephalies are a rare, genetically and clinically heterogeneous group of disorders that result from insufficient production of mature neurons during neurogenesis. Known causal genes, e.g. *ASPM* or *CEP152*, are involved in cell cycle control (DNA integrity check, DNA replication, centrosome duplication and spindle pole organization). Alterations of these processes may lead to isolated primary microcephaly (MicroCephaly Primary Hereditary, MCPH) or primary microcephaly with dwarfism (Seckel, MOPD2 and Meier-Gorlin syndromes) [1, 2]. In line with being caused by insufficient production of mature neurons before birth, and contrary to secondary microcephalies, primary microcephalies typically have a prenatal onset and present with a congenitally small brain, progressing after the first year of life to a fronto-occipital circumference smaller than 3 standard deviations below the mean for age and sex [3].

AP4M1 is a component of the Adaptor Protein Complex-4 (AP4). Homozygous mutations in *AP4M1* have been associated with severe intellectual disability, progressive spastic paraplegia, and inability to walk, in six unrelated families [4–7]. The AP4M1 defect shares these features with the defects of the other components of the heterotetrameric AP4 complex, namely AP4B1, E1 and S1, which have further been associated with short stature and, inconsistently, with microcephaly. Short stature and severe congenital microcephaly have however not been reported with AP4M1 mutation.

We here report on a patient with an *AP4M1* mutation, short stature, and severe, congenital microcephaly that initially mimicked MCPH.

## Case presentation

**Fig. 1:**
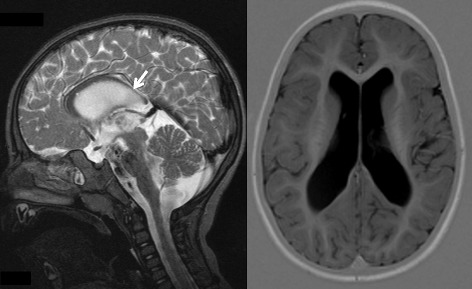
Brain MRI of the proband at age 2 years and 8 months. Note the normal cortex, temporo-parietal subcortical atrophy bilaterally, enlarged lateral ventricles, the thin corpus callosum predominantly in its caudal portion (*arrow*)

The patient was a male infant, first born after a 37 weeks gestation to second-cousin, asymptomatic parents of Turkish origin. Both parents had unremarkable medical histories, a normal head size and a normal intellect. They reported no paresis, and showed no spasticity nor hyperreflexia. The pregnancy was unremarkable, with no report of alcohol use or substance abuse, nor infection. Severe microcephaly was noted at birth, with a head circumference (HC) of 29 cm (−4.3SD), a length of 45 cm and a weight of 2.31 kg. Microcephaly progressed, with a HC of 41.5 cm at 1.1 year (−4.1SD), 43 cm at 3yo (−4.5SD), and 44 cm at 5.2 years (−4.7SD). The patient also had short stature, with a length at −3.5SD and a weight at −2.6SD. No dysmorphia was present except for clinodactily of the fifth fingers. Global hypotonia was present in the first months of life. Hypertonia was first noted at age 9 months, and progressed, with a bilateral Babinski sign. Partial complex epilepsy appeared at age 2 years and was treated successfully with valproate and lamotrigine. Motor development was severely impaired. The patient sat without support at age 1 year, crawled at 2 years, and stood with support at age 2.5 years. He walked with support after age 3 years. He had mild clubfeet deformities with flattened arches. Intellectual development was severely impaired. Testing at the age of 4 months revealed a developmental stage corresponding to the age of 3 months, and at the age of 9.5 months corresponding to 5 months (Bayley scales of infant and toddler development second edition). At the age of 8 years he had no words, but showed emotions. MRI at age 2y8m showed a normal cortex, temporo-parietal subcortical atrophy and hippocampal atrophy bilaterally, enlarged lateral ventricles, a thin corpus callosum predominantly in its caudal portion, a normal cerebellum and a normal white matter in FLAIR sequence acquisition (Fig. 1). A standard karyotype was normal 46,XY. Plasma amino acids and urine organic acids chromatograms showed normal patterns. The parents divorced and the mother had two other healthy children with a new partner.

After paediatric neurology evaluation and follow up, the patient was referred to the genetics clinic for testing of primary microcephaly genes. In a first round of analysis, we used a 250 K SNP GeneChip® microarray (AffymetrixTM, Inc.) and found heterozygosity of alleles at loci MCPH1,2,3,4,5,7 and 9, making the corresponding genes unlikely to cause microcephaly in the proband. Direct sequencing of the coding exons and exon-intron junctions of genes *ASPM* and *WDR62* showed no abnormality.

For whole-exome sequencing, the proband’s genomic DNA was sheared and exonic sequences enriched using Roche SeqCap EZ Human Exome v3.0 (64 Mb) DNA capture. Sequencing was performed on a HiSeq1500 Illumina sequencer at the BRIGHTcore BRussels Interuniversity Genomics High Throughput core [8]. Raw sequences were aligned to the reference genome GRCh37 using BWA algorithm version 0.7.10 [9], duplicated reads were then marked using Picard version 1.97 [10], alignment quality was improved using the GATK [11] realigner and base recalibrator version 2.7, and finally, variants were called using GATK Haplotype Caller version 2.7. The resulting variant set was annotated and filtered using the Highlander software [12]. Variants were filtered for quality criteria (pass GATK standard filter, read depth > 5, variant confidence by depth ≥ 10), allelic frequency < 0.5% (based on the maximum minor allele frequency found in ExAC, 1000G, ESP6500, gonl, ARIC5606 and our in-house database), nonsynonymous or splice junction effect in protein coding genes (using biotype from Ensembl [13] and snpeff_effect from SnpEff [14]) and genotype (homozygous or heterozygous variants in a subset of 68 primary microcephaly genes that we extracted from the literature (Additional file 1), and homozygous or biallelic variants from all other genes of the exome). Variants were then sorted by decreasing Combined Annotation Dependent Depletion (CADD) score [15]. A CADD score between 0 and 10 is associated with non-deleterious variants, and scores greater or equal to 20 are associated with the 1% most deleterious substitutions possible. The variant of interest was confirmed by Sanger sequencing of exon 13 of *AP4M*1. DNA was amplified using a standard Polymerase Chain Reaction (forward primer: AGTACAGCCCACACCCACAC, reverse primer: CACCTTCTTGAGGCAGACCC). The PCR product was purified with Exosap-IT (Affymetrix), and sequenced by the company Beckman Coulter Genomics.

**Fig. 2:**
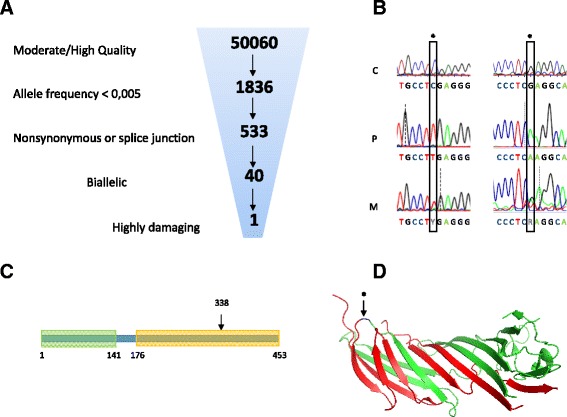
AP4M1 mutation and domains. **a** Exome sequencing data from proband, showing the filtering parameters used to sort through the variant dataset. **b** Sanger sequencing of part of exon 13 of the AP4M1 gene, forward (*left*) and reverse (*right*). The C to T mutation (*) at position 1170 of the coding DNA sequence was found homozygous in the proband (P), and heterozygous in his mother (M); a normal sequence is shown in an unrelated control subject (C). **c** Linear presentation of the AP4M1 protein, showing the Longin-like domain (residues 1–141) and Mu homology domain (residues 176–453). *Arrow*, position of the Arg338X mutation. **d** Crystal structure (PDB 3 l81 [27]) of AP4M1 C-terminal domain (residues 160–453). The portion of the protein truncated by the mutation appears in *red*

The affected child’s exome sequence data were first analyzed for rare (allele frequency <0.5%) variants in 68 primary microcephaly-related genes. This showed heterozygous missense variants in three genes: *ATR* (c.6109A > G p.Met1996Thr), *MCPH1* (c.85C > T p.Ala3Ala) and *BLM* (c.3967G > A p.Gln1283Gln). Analysis of the rest of the exome data revealed 5 hemizygous, 9 compound heterozygous, and 26 homozygous variants, two of which being encompassed by a 9 Mb homozygous stretch (chr7: 91599825–100494222), consistent with homozygosity by descent (autozygosity) for this chromosomal segment. One of these three variants, located at Chr7:99,703,901, consisted of a homozygous truncating mutation in exon 13 of the *AP4M1* gene, c.1170C > T (transcript_uniprot_id O00189) changing the Arginine at position 338 of the polypeptide into a stop codon, p.Arg338X (Fig. 2). The variant frequency was 3.31×10^(−5) in the Exome Aggregation Consortium [16] with only four alleles reported, all heterozygous, in 1 Latino and 3 European subjects. The variant was absent from 1000G, GoNL, ESP, and our in-house database. The mutation was located in the middle of the predicted Mu homology domain of AP4M1 which spans amino acid residues 176 through 453 (Fig. 2, left panel). Sanger sequencing confirmed homozygosity of the mutation in the proband and heterozygosity in both parents, as shown in Fig. 2 right panel (father not shown). Among all variants observed in the patient, this was the only variant predicted to cause a premature termination codon. It yielded a CADD score = 39, the highest of all observed variants.

## Discussion and conclusions

We investigated a child initially referred for primary microcephaly of prenatal onset. Exome sequencing revealed only one rare (MAF < 0.5%) variant predicted to introduce a premature termination codon. This mutation was located in exon 13 of gene *AP4M1* which has 15 exons, and caused the replacement of an arginine by a stop codon at position 338 (p.Arg338X). It is predicted to truncate 41% of the predicted Mu homology domain of the AP4M1 protein. This domain is an important protein-protein interaction module found in proteins involved in endocytosis. It has been shown to interact with the cytosolic tail of transmembrane cargo proteins, through binding to tyrosine-based signals [17–20]. Either this truncation, or nonsense-mediated decay of the entire mRNA, is very likely to cause complete loss of AP4M1 function in the proband.

AP complexes are important for vesicular trafficking from the trans-golgi network to the plasma membrane [21], with specificity of the AP complexes in selection of the vesicles trafficked to or from the membrane and inclusion of molecules to be cargoed in the vesicle [22]. AP4B1, E1 and S1 are, like M1, subunits of the AP4 complex, a heterotetramer. Defects of any of the four subunits have been found in patients with autosomal recessive intellectual disability and progressive spastic paraplegia. These features were present in the proband, providing further evidence for the causality of the Arg338X mutation.

The proband presented with microcephaly of prenatal onset and developed a short stature, features which have not been reported in other patients with AP4M1 mutations, including homozygous truncating mutations, making a more severe functional effect of the mutation in the present proband unlikely to explain the more severe phenotype. Interestingly, exome analysis also showed heterozygous variants in three genes, *ATR*, *MCPH1* and *BLM*, which are known causes of autosomal recessive primary microcephaly, presumably via their role in DNA damage repair [23], homozygous mutation of either of these genes causing increased apoptosis of neural progenitors [24]. *IER3IP1* is another gene whose defect causes microcephaly via increased apoptosis of neural progenitors [25]. It encodes a small polypeptide localized at the ER and Golgi which, like the AP4M1 product, plays a role in vesicle trafficking. It is thus tempting to speculate that the additional heterozygous variants in *MCPH1*, *ATR* and *BLM*, together modified the effect of the homozygous *AP4M1* mutation, causing increased apoptosis of neural progenitors, and perhaps of other cell types. Of note, our patient also had short stature, a feature reported with homozygous mutations of either *MCPH1*, *ATR*, or *BLM* ([23] and references therein).

Additional file 2 provides a compilation of clinical features in all reported cases of AP4 defects and in the present proband. While postnatal microcephaly, mild to moderate (−1SD to −4 SD) has been reported in patients with AP4B1, E1 and S1, all had normal head circumferences at birth, except one sibship (family ID01) where microcephaly was reported as present since birth albeit with no measures reported [26]. In addition, severe microcephaly (<−4SD), and, more importantly, of prenatal onset, has not been reported with AP4M1 mutations.

Our findings associate the AP4M1 mutation with severe microcephaly of prenatal onset and short stature, and suggest increased apoptosis as a likely mechanism. This is important for the genetic diagnosis of congenital microcephaly using massive parallel sequencing of multigene panels, or clinical exomes. More generally, our findings suggest that the AP4 mutation-related microcephaly shares mechanisms of prenatal neural progenitor depletion with other causes of congenital, primary microcephaly.

## Additional files

Additional file 1: 68 primary microcephaly-associated genes used for initial analysis of whole exome sequencing data. (PDF 34 kb)

Additional file 2: Clinical features in reported cases of AP4 defects and in the present proband. (PDF 76 kb)

## Abbreviations

AP4: Adaptor protein complex-4
HC: Head circumference
MCPH: MicroCephaly primary hereditary
PCR: Polymerase chain reaction
