# Supplementary material for: Severe congenital microcephaly with AP4M1 mutation, a case report
Source: BMC Med Genet. 2017 May 2;18:48. doi: 10.1186/s12881-017-0412-9 (PMC5414176; doi:10.1186/s12881-017-0412-9)
Supplement: Supplementary file 1 — 68 primary microcephaly-associated genes used for initial analysis of whole exome sequencing data. (PDF 34 kb) [file 12881_2017_412_MOESM1_ESM.pdf]

ARHGAP11B	CEP250	MYCN	SLC25A19
ASPM	CEP63	NBN	SLC9A6
ATR	CIT	NDE1	SMC1A
ATRIP	COX7B	NIN	SMC3
BLM	DYRK1A	NIPBL	STAMBP
BRAT1	EFTUD2	ORC1	STIL
BUB1B	EIF2AK3	ORC4	TRMT10A
CASC5	ERCC3	ORC6	TUBA1A
CASK	ERCC4	PCNT	TUBB
CCDC7	ERCC5	PHC1	TUBB2B
CDC6	ERCC6	PLK4	TUBB3
CDK5RAP2	ERCC8	PNKP	TUBG1
CDT1	IER3IP1	PPM1D	TUBGCP4
CENPF	KIF11	RAD50	TUBGCP6
CENPJ	KMT2B	RBBP8	UBE3A
CEP135	LIG4	RNU4ATAC	WDR62
CEP152	MCPH1	SASS6	ZEB2

Additional file 1: 68 primary microcephaly-associated genes used for initial analysis of whole exome sequencing data
