# Supplementary material for: Severe congenital microcephaly with AP4M1 mutation, a case report
Source: BMC Med Genet. 2017 May 2;18:48. doi: 10.1186/s12881-017-0412-9 (PMC5414176; doi:10.1186/s12881-017-0412-9)
Supplement: Supplementary file 2 — Clinical features in reported cases of AP4 defects and in the present proband. (PDF 76 kb) [file 12881_2017_412_MOESM2_ESM.pdf]

	**Gene Mutation**	**number of affected children**	**OFC at birth**	**OFC after age 1 yr**	**walk with /without support**	**short stature**	**mental retardation**	**language**	**stereotypic laugh**	**shy character**	**neonatal hypotonia**	**progressive hypertonia +contractures**	**hyperreflexia**	**spasticity**	**seizures**	***facial features***	**other features**	**adducted thumbs**	**MRI**
**Abou Jamra /Colleaux 2011**	AP4B1 p.Glu163_Ser739delinsVal	3	microcephaly	-2, -2.5, -3 SD	inability to walk without aid	yes	severe	marked speech delay	yes	yes	yes	yes	yes	spastic paraplegia	no	*high palate, mildly remarkable facial gestalt with a wide nasal bridge*	hyperlaxity, genu recurvatum, pes planus, and a waddling gait		
**Abou Jamra /Colleaux 2011**	AP4S1 p.Arg42*	3	?	-1, -4, -2 SD	walked at age 2 but lost ability to walk 6-24 months later	yes	severe	absent	kept smiling or laughing for no obvious reason	yes	yes	yes, especially lower limbs	2/3 yes	mild spasticity in flection of upper limbs, which could be used only for simple tasks	no	*prominent and bulbous nose, a wide mouth, and coarse features*	talipes equinovarus; weak and decreased muscle mass of the shanks		
**Abou Jamra /Colleaux 2011**	AP4E1 p.Glu181Glyfs*20	2	?	-3, -4 SD	crawling	yes	severe	absent	kept smiling or laughing for no obvious reason	yes	yes	yes, especially lower limbs	?	yes	1/2 yes	*prominent and bulbous nose, a wide mouth, and coarse features*	talipes equinovarus; and decreased muscle mass of the shanks		
**Abdollahpour /Kutsche 2015**	AP4B1 2-bp deletion	2	34 cm (gestational age 41 weeks)	-2 SD	Patient1: short distances at 4yr, wheelchair since 12yr. Patient2: without aid until 7,5yo, with aid until 12yr, then wheelchair	yes -2 SD	severe	Patient1: short sentences. Patient2: absent.	no	yes		increased tonus of lower limbs	yes	spastic tetraplegia with contractures, particularly lower limbs	Patient2: yes.	*tongue protrusion, open mouth, widely spaced teeth, gingival hyperplasia, prominent supraorbital ridges, and a broad nasal root*	Patient1: club foot, skin hyperpigmentation on the lumbar region, and low anterior and posterior hairline. Patient2: thoracic dextroscoliosis, and lumbar levoscoliosis.		Patient1: normal. Patient2: mild thinning of the corpus callosum in the dorsal region of the splenium.
**Jameel /Dahl 2014**	**AP4M1 p.Y65Ffs*50**	**2**	normal	-2 SD	Patient1: walk without support at 2yr, lost at 6yr. Patient2: walk at 4yr, lost at 6yr.	no	severe	few words		agressive behaviour		contractures of feet		lower limbs	yes	*Patient2: short philtrum and a bulbous nose.*			Patient1: thin posterior corpus callosum, an enlarged third ventricle and widened temporal horns of the lateral ventricles. The mesencephalon and pons were thinner than expected for age. The upper vermis was hypoplastic/atrophic.
**Langouet /Colleaux 2015**	**AP4M1 c.1137+1G>T**	**2**	normal	-1.5, -2 SD	Patient1: walked with support from 15mo to 4yr. Patient2: never walked.		severe	few words	Patient2: yes.		yes		yes	yes			columella, short neck		Patient1: cerebral atrophy. Patient2: partial agenesis of the corpus callosum with a lipoma as well as delayed myelinization.

**Verkerk /Mancini 2009**	**AP4M1 c.1137+1G/T**	**5**	?	-1, 0, -2, ?, -2.5 SD	never achieved		severe	almost absent	yes		yes	yes		yes			strabismus	yes	abnormal WM, wide ventricles, 2/3 cerebellar atrophy
**Tuysuz /Gunel 2013**	**AP4M1 Arg338Ter**	**2**		-3, -2 SD	Patient1: walked with support from 3yr to 4yr. Patient2: walked with support from 2,5yr to 4yr.		severe	few words	yes			yes		spastic tetraplegia	yes			no	asymmetrical ventriculomegaly, thin splenium of the corpus callosum, abnormal white matter changes, and hippocampus globoid formation, flat and thin hippocampus on the right side
**Tuysuz /Gunel 2013**	**AP4M1 p.Arg318Ter**	**2**		-4,-2 SD	never walked		severe	almost absent	yes			yes		spastic tetraplegia	yes		Patient2: strabismus.	yes in 1/2	asymmetrical ventriculomegaly, thin splenium of the corpus callosum, abnormal white matter changes, and hippocampus globoid formation
**Tuysuz /Gunel 2013**	AP4B1 p.L221fs	2		-1.5, -1.5 SD	Patient1: walk with support. Patient2: walked without support from 3yr to 5yr.		severe	few words	yes		yes	yes		spastic tetraplegia	yes			no	asymmetrical ventriculomegaly, thin splenium of the corpus callosum, abnormal white matter changes, and hippocampus globoid formation, flat and thin hippocampus bilaterally
**Present patient**	**AP4M1 p.Arg388***	**1**	-4.3 SD (gestational age 37 weeks)	-4.7 SD (5.2yo)	walk with support	yes (lenght -3.5 SD, weight -2.6 SD)	severe	babbles, no words	no	no	yes	yes	yes		yes	*no*	clinodactyly both little fingers, flattened arch, mild clubfeet, mild syndactily 2nd and 3rd toe, short toes, all of same length except shorter 5th toe	no	rather large ventricles, especially the lateral ventricles and to a lesser extent also the third and fourth ventricle

Additional file 2: Clinical features in reported cases of AP4 defects and in the present proband
